# Ascorbic Acid Enhances the Accumulation of Polycyclic Aromatic Hydrocarbons (PAHs) in Roots of Tall Fescue (*Festuca arundinacea* Schreb.)

**DOI:** 10.1371/journal.pone.0050467

**Published:** 2012-11-21

**Authors:** Yanzheng Gao, Hui Li, Shuaishuai Gong

**Affiliations:** 1 Institute of Organic Contaminant Control and Soil Remediation, College of Resource and Environmental Sciences, Nanjing Agricultural University, Nanjing, P. R. China; 2 Department of Crop and Soil Sciences, Michigan State University, East Lansing, Michigan, United States of America; Indian Institute of Toxicology Research, India

## Abstract

Plant contamination by polycyclic aromatic hydrocarbons (PAHs) is crucial to food safety and human health. Enzyme inhibitors are commonly utilized in agriculture to control plant metabolism of organic components. This study revealed that the enzyme inhibitor ascorbic acid (AA) significantly reduced the activities of peroxidase (POD) and polyphenol oxidase (PPO), thus enhancing the potential risks of PAH contamination in tall fescue (*Festuca arundinacea* Schreb.). POD and PPO enzymes *in vitro* effectively decomposed naphthalene (NAP), phenanthrene (PHE) and anthracene (ANT). The presence of AA reduced POD and PPO activities in plants, and thus was likely responsible for enhanced PAH accumulation in tall fescue. This conclusion is supported by the significantly enhanced uptake of PHE in plants in the presence of AA, and the positive correlation between enzyme inhibition efficiencies and the rates of metabolism of PHE in tall fescue roots. This study provides a new perspective, that the common application of enzyme inhibitors in agricultural production could increase the accumulation of organic contaminants in plants, hence enhancing risks to food safety and quality.

## Introduction

Polycyclic aromatic hydrocarbons (PAHs) are persistent organic pollutants (POPs) that demonstrate carcinogenic and mutagenic toxicities [1], [2]. These contaminants are frequently detected at relatively high concentrations (mg/kg) in soils worldwide [3], [4]. Plants can absorb these contaminants from soil, causing deleterious effects on human and animal health via the consumption of contaminated vegetables [5]–[7]. Therefore, an improved understanding of plant uptake of PAHs is essential for assessment of both the exposure of humans and other animal species and the risk represented by PAH-contaminated sites.

Recently, plant uptake of organic contaminants such as PAHs has attracted considerable attention [6]–[11]. PAHs enter plants via foliage uptake from the atmosphere [1], [3] and root uptake from contaminated soil [12], [13]. Gao and Collins (2009) quantified the contributions of these two PAH uptake pathways in white clover. A significant fraction of shoot contamination resulted from the aerial deposition of volatilized PAHs, particularly of compounds with log K_OA_>9 and log K_AW_<−3 (K_OA_: octanol–air partition coefficient, K_AW_: dimensionless air–water partition coefficient) [2]. PAH uptake by plants from the soil to roots is a major pathway; the subsequent transport to shoots via the transpiration stream flux favors compounds with greater aqueous solubility [6], [14]. The magnitude of root uptake depends primarily on the lipid contents of plant roots, which is itself dependent on the protein, fat, nucleic acid, and cellulose contents; these contain lipophilic components and serve as the major domains accommodating PAHs after penetration of plant root surfaces. Recently, Kang *et* *al*. (2010) reported that the lipid contents of intracellular components determined the accumulation of lipophilic compounds; *e*.*g*. PAHs, and that the corresponding diffusion rate was determined by the concentration gradient between the cell wall and intracellular organelles [7].

Many organic chemicals, including PAHs, are metabolized by plants, and exhibit a reduced concentration in plant tissues [11], [15], [16]. However, these metabolic processes have been studied for only limited types of organic contaminants; for example, trichloroethylene, benzene, explosives and herbicides [17]–[19]. The metabolic processes vary according to the type of contaminant and plant species. However, little information is available on the metabolism of PAHs by plants [15], [16]. A recent study showed that phenanthrene (PHE) was metabolized into other polar products in *Zea mays* [20]. In another study, anthracene (ANT) was metabolized primarily in cell walls, and the formed products were bound to cell wall components such as pectin, lignin, hemicellulose, and cellulose [16], [21].

**Table 1:** Selected physicochemical properties of the PAHs used in this study [33].

PAHs	Molecular mass (g/mol)	log K_ow_	S_w_ (mg/L)^a^	K_H_ (unitless)
Naphthalene	128.17	3.37	31.0	1.74×10^−2^
Phenanthrene	178.23	4.57	1.1	1.31×10^−3^
Anthracene	178.23	4.54	0.045	1.60×10^−3^

a at 25°C.

Metabolic processes are controlled by a variety of enzymes. For instance, cytochrome P450 monooxygenase could detoxify herbicides such as fenoxaprop-ethyl, diclofop-methyl, and bentazon in plants [22], [23]. Polyphenol oxidase (PPO), commonly found in fungi and plants, refers to a group of enzymes that catalyze the oxidation of phenolic compounds [24]. Peroxidase (POD), another type of oxidative enzyme commonly present in plant and animal tissues, can oxidize phenols and aromatic amines in the presence of hydrogen peroxide. In contrast, the oxidation of phenolic compounds by PPO requires the presence of oxygen gas [25]. Both PPO and POD play important roles in the metabolism of aromatic compounds in soil and water [26], [27]. However, little information is available regarding their function in the metabolism of PAHs by plants.

**Figure 1:**
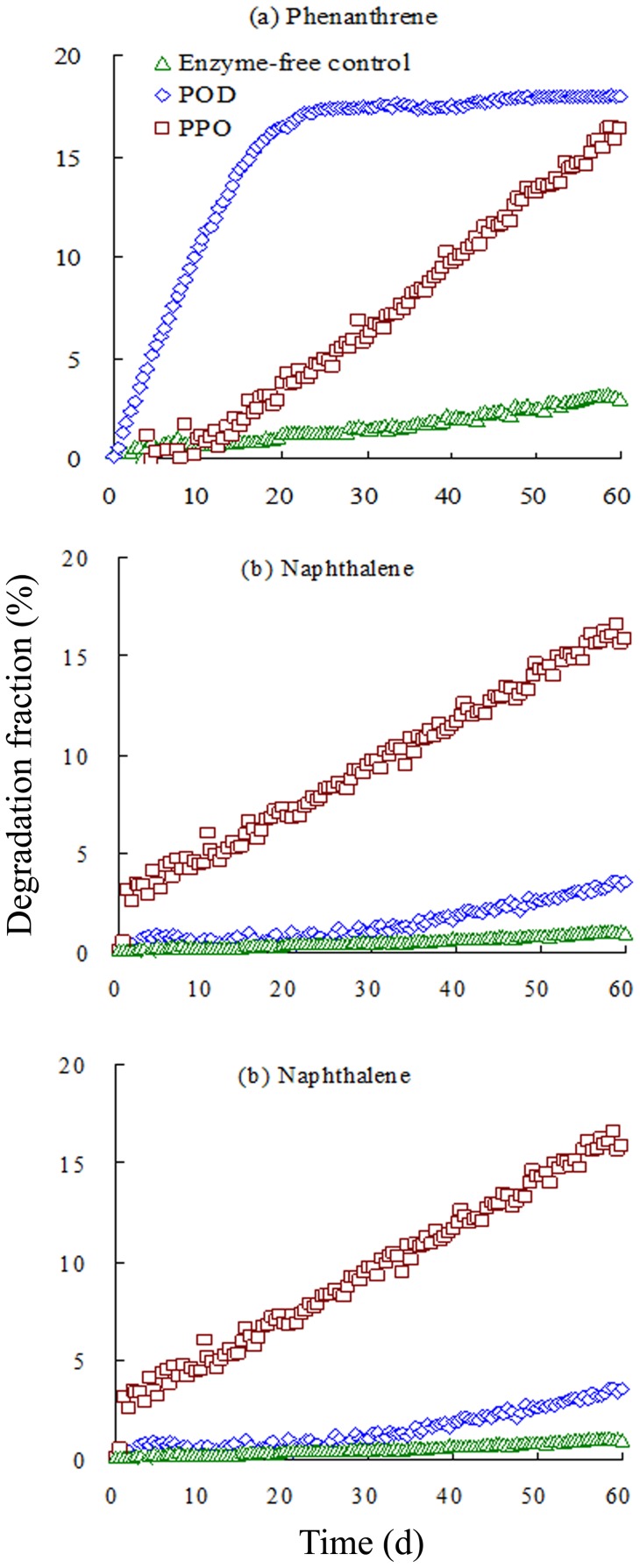
In vitro degradation fraction (%) of added (**a**) phenanthrene, (b) naphthalene, and (**c**) anthracene by POD and PPO over time The degradation fraction (D_PAH_, %) of PAHs was estimated as: D_PAH_  =  (C_PAH-o_ – C_PAH-i_)/C_PAH-o_×100%, where C_PAH-i_ and C_PAH-o_ represent the PAH concentrations in solution at time i and time 0, respectively.

Inhibitors are commonly utilized in agricultural production to control enzyme activities and the metabolism of organic components, such as herbicides, by plants. Sterling and Balke (1990) reported reduced effects of monooxygenase inhibitors (1-aminobenzotriazole, tetcyclasis, piperonyl butoxide and cinnamic acid) on the oxidative metabolism of bentazon in rice and in soybean cell cultures [28]. This inhibition has also been reported for the herbicides fenoxaprop-ethyl and diclofop-methyl in wheat or barley by tetcyclacis and tridiphane [23]. Gronwald and Connelly (1991) reported that the cytochrome P450 inhibitor phenylhydrazine significantly diminished bentazon metabolism; moreover, other inhibitors–such as 3(2,4-dichlorophenoxy)-1-propyne and aminobenzotriazole–also reduced bentazon metabolism, albeit to a lesser extent [22]. A significant decrease in the activities of arginine decarboxylase and ornithine decarboxylase occurred when DL-α-difluoromethylornithine (DFMO) and DL-α-difluoromethylarginine (DFMA) were added to a maize (*Zea mays* L.) callus culture medium, and resulted in irreversible inhibition of putrescine synthesis [29]. However, to our knowledge, most previous studies of the effects of enzyme inhibitors on plant metabolism focused primarily on herbicide applications in agricultural production, and little is known about the effects of inhibitors on plant metabolism of absorbed POPs such as PAHs.

**Figure 2:**
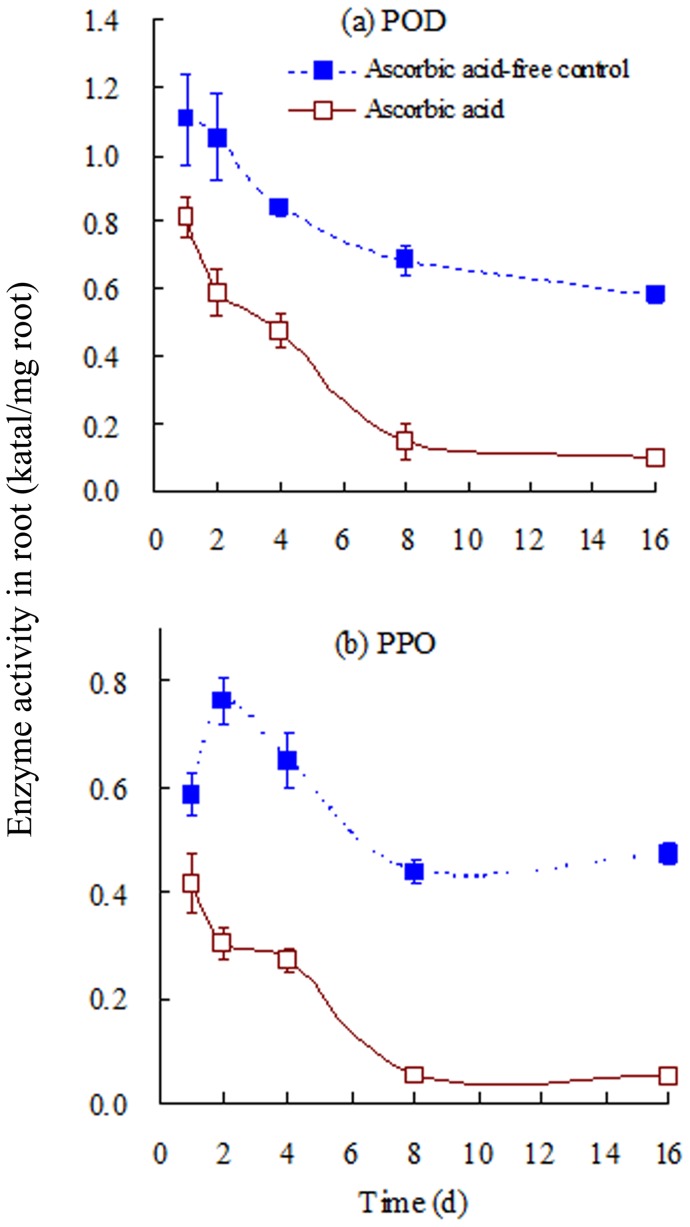
(a) POD and (b) PPO activities in the roots of tall fescue over time. The uptake time was defined as the time frame from the immersion of plant roots in solution containing phenanthrene to the removal of plant for extraction. Error bars represent standard deviation (SD). The initial phenanthrene concentration was 1.0 mg/L.

Ascorbic acid (AA) is a naturally occurring, water-soluble compound with desirable characteristics as an enzyme inhibitor. It is the most abundant antioxidant in plants, and is used in agriculture to enhance plant stress-resistance [30]. A recent *in vitro* study reported that AA inhibits the activity of PPO in *Mangifera indica* L. [25]. However, few studies have investigated the effects of AA on enzyme activities and the metabolism of PAHs by plants.

**Figure 3:**
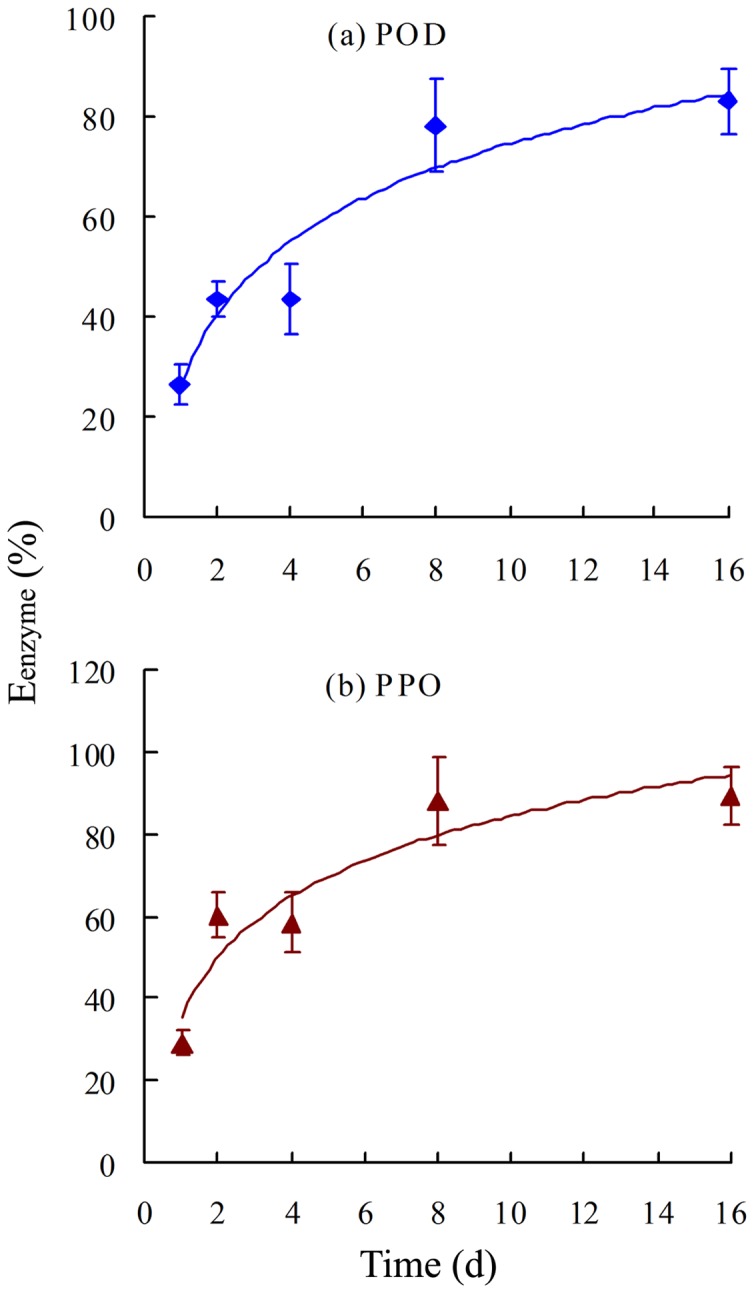
Inhibition efficiency (E_enzyme_, %) of ascorbic acid on (a) POD and (b) PPO activities in plant roots. E_enzyme_ was calculated as: E_enzyme_  =  (U_c_–U_AA_)/U_c_×100%, where U_AA_ and U_c_ represent the enzyme activities in roots grown in the presence and absence of AA, respectively. Error bars represent standard deviations (SD).

To this end, the objective of this study was to evaluate the influence of the commonly used inhibitor, AA, on plant enzyme activities and PAH uptake. Naphthalene (NAP), PHE, and ANT, as representative 2- and 3-ringed PAHs, were the PAHs used. Tall fescue (*Festuca arundinacea* Schreb.) is a common pasture plant for livestock production, and is also used in phytoremediation due to its fibrous root system and large root-specific surface area [5]. In addition, the uptake of PAHs by this plant has been reported [31], [32]. Hence, tall fescue was chosen as a test plant in this investigation. These findings suggest that the common use of enzyme inhibitors in agricultural production may promote the accumulation of organic contaminants in plants, hence increasing risk in terms of food safety and quality.

**Figure 4:**
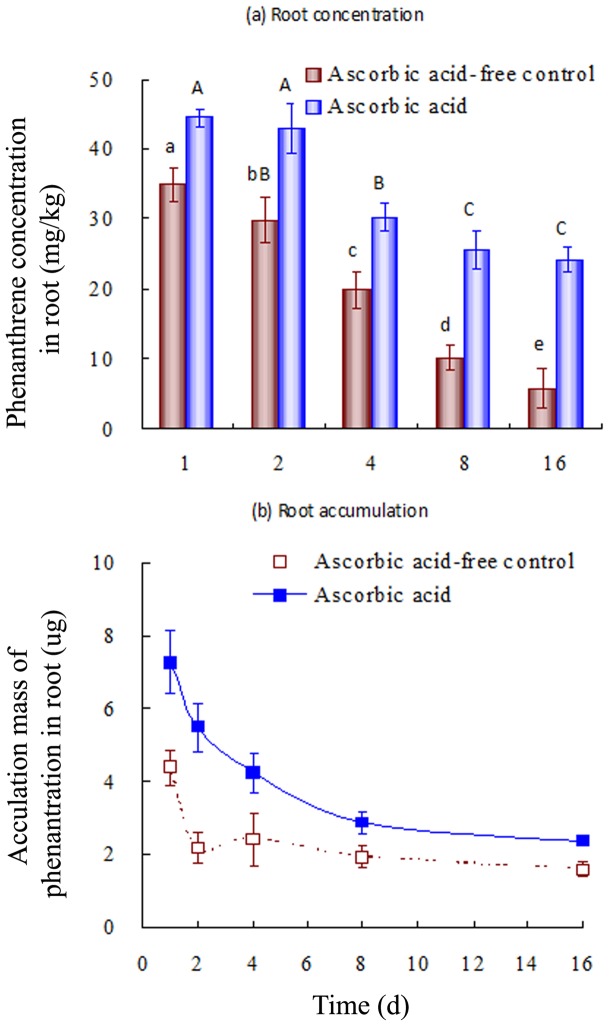
(a) Phenanthrene concentration and (b) accumulation in the roots of tall fescue as a function of uptake time. The initial phenanthene concentration was 1.0 mg/L. Error bars represent standard deviations (SD). Values above columns followed by the same letter are not significantly different (*p*>0.05).

## Materials and Methods

### Reagents

NAP, PHE, and ANT at purities of >97% were purchased from Aldrich Chemical Company. Their physicochemical properties are listed in Table 1 [33]. POD and PPO were purchased from Shanghai Kayon Biological Technology Co. Ltd., and were of BR grade and exhibited activities of >0.050 and 0.083 katal/mg, respectively. Other chemicals used were of analytical grade.

**Figure 5:**
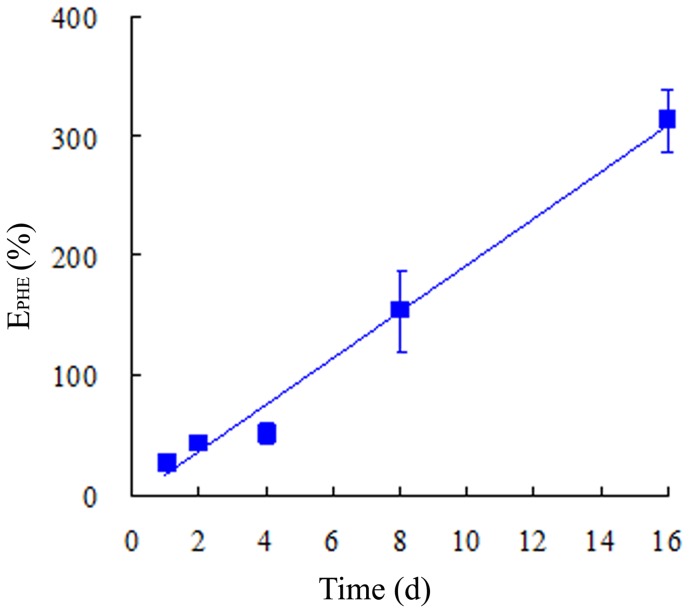
Inhibition efficiency (E_PHE_, %) of ascorbic acid on phenanthrene degradation in plant roots as a function of time. E_PHE_ was calculated as: E_PHE_ (%)  =  (C_PHE-AA_–C_PHE-c_)/C_PHE-c_×100%, where C_PHE-c_ and C_PHE-AA_ represent the phenanthrene concentrations in plant roots grown without and with AA, respectively. Error bars represent standard deviations (SD).

### *In vitro* degradation of PAHs by POD and PPO

POD and PPO stock solutions with enzyme activities of 0.15 katal/mL were prepared by dissolving POD and PPO in PBS and Tris-HCl buffers, respectively. PAHs were degraded *in vitro* by mixing 1.0-mL POD or PPO stock solution with 9.0-mL PAH solution. The final concentrations of NAP, PHE, and ANT were 10, 1.0 and 0.04 mg/L, respectively [32], [34]. Control treatments were conducted using the same amounts of PBS or Tris-HCl buffer and PAHs but in the absence of POD or PPO enzymes. The concentration of PAHs in solution was determined by fluorescence density measurement using an enzyme-labeled instrument (SpectraMax M5, Molecular Devices). The excitation and emission wavelengths were 260 and 340 nm for NAP, 280 and 340 nm for PHE, and 250 and 370 nm for ANT, respectively [35].

**Figure 6:**
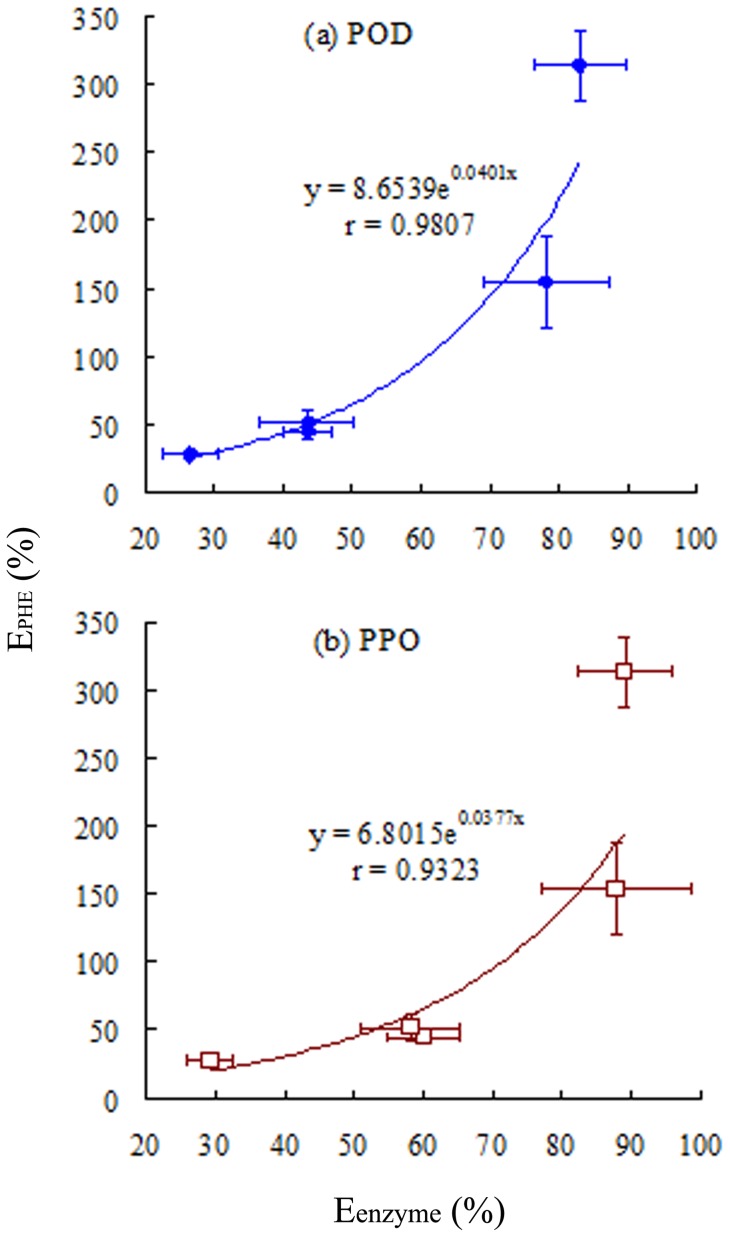
Relationship between E_PHE_ and E_enzyme_ for (a) POD and (b) PPO. E_PHE_ and E_enzyme_ are the inhibition efficiencies of ascorbic acid on phenanthrene degradation and enzyme activities in plant roots, respectively. E_PHE_ was calculated as: E_PHE_ (%)  =  (C_PHE-AA_–C_PHE-c_)/C_PHE-c_×100%, where C_PHE-c_ and C_PHE-AA_ represent the phenanthrene concentrations in plant roots grown without and with AA, respectively. E_enzyme_ was calculated as: E_enzyme_  =  (U_c_–U_AA_)/U_c_×100%, where U_AA_ and U_c_ represent the enzyme activities in plant roots grown in the presence and absence of AA, respectively. Error bars represent standard deviations (SD).

**Figure 7:**
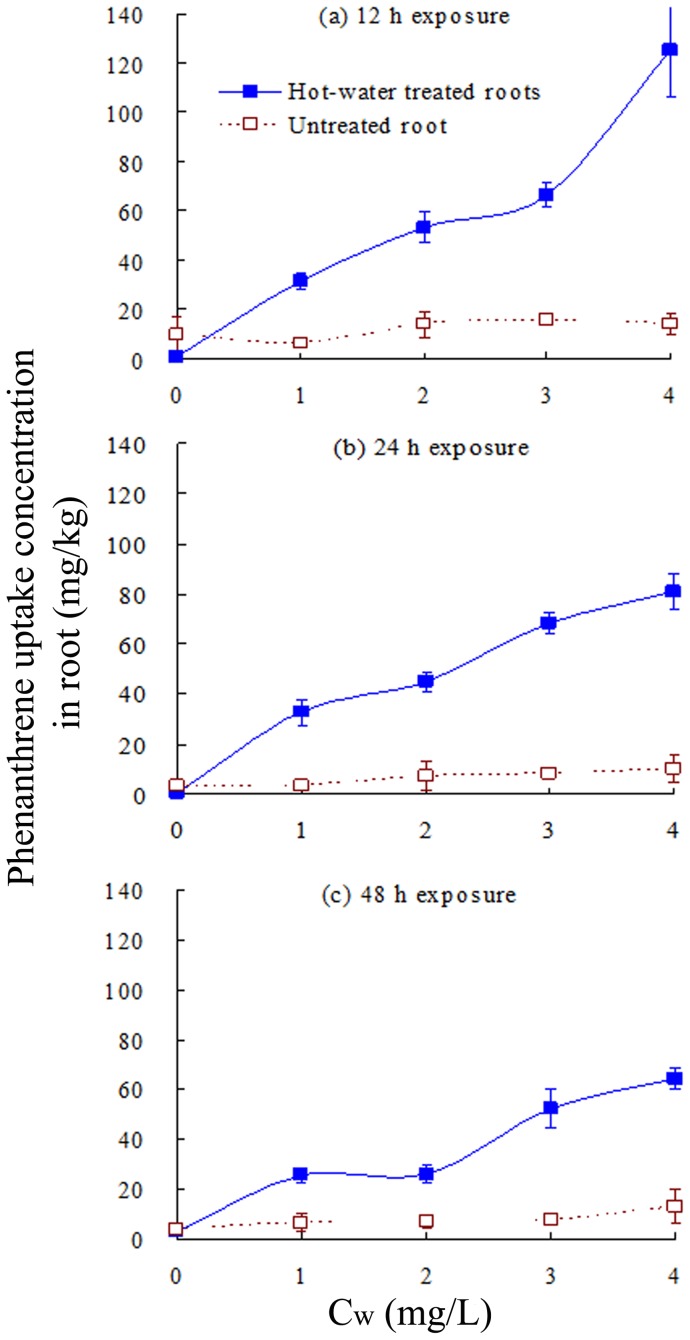
Phenanthrene uptake (mg/kg) in tall fescue roots treated by immersion in 100°C water for 5 min, compared to the control. Plant roots were exposed to phenanthrene at initial concentrations of 0–4 mg/L in aqueous solution and sampled after (a) 12 h, (b) 24 h, and (c) 48 h. Error bars represent standard deviations (SD).

### Plant uptake of PHE from water with/without AA

Tall fescue (*Festuca arundinacea* Schreb.) was selected for the uptake of PHE using a batch method [5], [8], [10], [32]. After seeds germinated on vermiculite, seedlings were transferred to a tray containing half-strength Hoagland solution.^5^ The seedlings were allowed to grow in a greenhouse for 2 weeks at 25–35°C during the daytime, and at 15–25°C during the night with a relative humidity of 60–80% until the plants reached ∼15 cm in height and developed relatively mature roots. These plants were then utilized in the PHE uptake experiment.

A group of 12 tall fescue plants was loosely bound together at the lower part of the plant shoots using Teflon tape, and cultured in an amber glass container through a drill hole in the cap. Plant roots were immersed just below the level of half-strength Hoagland solution and cultured hydroponically for 3 days. The culture solution was then replaced with the same Hoagland solution containing 1.0 mg/L PHE and/or 2.0 mg/L AA. The control treatments were prepared in the same manner but without AA. Four replicates of each treatment were performed. Solution losses from the containers due to plant uptake and evaporation were weighed using a balance on a routine basis during the study period. Lost solution was replenished with the same amount of solution, which was taken from control containers that contained plants in PHE-free growth media. After plant uptake for 1, 2, 4, 8, and 16 days, the plants were sampled and prepared for analyses of PHE concentration and enzyme activities. The uptake time was defined as the time elapsed between the addition of the solution containing PHE and removal of the plant for extraction.

### Analysis of PHE in plant roots

The detailed analytical procedure for detecting PHE in plant roots is referred to in Gao *et* *al*. (2010) [14]. In brief, seedling roots were washed several times using ultrapure water, freeze-dried and homogenized by grinding. The dried plant root residues were weighed and subjected to ultrasonication extraction in 1∶1 (v/v) acetone: hexane for 1 h. The extraction solvent was then collected and replenished with new acetone: hexane solvent, followed by a further 1 h ultrasonication extraction. This process was repeated three times to achieve satisfactory PHE recovery. The extracts were combined, evaporated and dissolved in hexane, followed by filtration through a 2-g silica gel column, and eluted using 11.0 mL of 1∶1 (v/v) hexane: dichloromethane. The samples were then evaporated, dissolved in 2.0-mL methanol, and analyzed by high-performance liquid chromatography (HPLC). Recovery of PHE spiked in plant roots averaged 103.4% (n = 5, RSD<3%) after being subjected to the entire extraction procedure.

### Analysis of POD and PPO enzyme activities in plants

The procedure used to determine POD and PPO enzyme activities was adapted from Hao *et* *al*. (2004) Ling *et* *al*. (2012), and Lu *et* *al*. (2008) [32], [34], [36]. Fresh plant roots (0.10 g) were mixed with 0.05-g polyvinylpyrrolidone (PVP) in 10.0-mL phosphate buffer, ground in an ice bath, and sieved through a nylon mesh. The pH of the phosphate buffer was adjusted to 7.8 for POD and 6.0 for PPO activity measurement. The above procedure was conducted at 4°C. For PPO analysis, 2.0 mL of 0.01 M phosphate buffer (pH 6.0), 1.0 mL of 0.1 M *o*-dihydroxybenzene, and 0.2 mL of the prepared filtrate were sequentially added to a 5.0-mL colorimeter tube. For POD analysis, 0.2-mL plant root extracts were mixed with 2.0 mL of 0.1 M acetate buffer (pH 5.0), 1.0 mL of 0.25% guaiacol and 0.1 mL of 0.75% H_2_O_2_ in colorimeter tubes, which were maintained at 37°C in a water bath for 15 min prior to measurement. Enzyme activities were determined by colorimetry at 420 nm for PPO and 470 nm for POD. One unit of enzyme activity was defined as the amount of enzyme that produced an increase in absorbance (OD) of 0.01/min at the selected wavelength.

### Statistical analyses

All data collected were processed using the Microsoft Excel software. Each data point is the mean of at least three replicates, and error bars represent standard deviations (SD). Data were statistically analyzed by analyses of variance (ANOVA) using the statistical package SPSS, version 13.0 (SPSS, Inc.). Differences were taken to be significant at a value of *p*<0.05.

## Results

### *In vitro* degradation of PAHs in the presence of POD and PPO

The *in vitro* degradations of NAP, PHE, and ANT in the presence of POD and PPO enzymes are shown in Figure 1, along with the enzyme-free controls for comparison. The degradation fraction (D_PAH_, %) of PAHs was estimated.



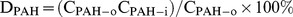
(1)where C_PAH-i_ and C_ PAH-o_ are the PAH concentrations in solution at time i and time 0. This *in vitro* experiment clearly demonstrated that the presence of POD and PPO in the solution facilitated the decomposition of PAHs with varying degradation rates. In the control treatments, the concentration of the test PAHs decreased by less than 3.1% during the experimental period, possibly due to abiotic dissipation processes such as sorption to glass walls and/or volatilization. PAH degradation in the presence of POD and PPO was significantly greater than that in the enzyme-free controls. Approximately 17.9% and 16.5% of PHE, 3.52% and 16.5% of NAP, and 34.7% and 16.3% of ANT were decomposed within 60 min, indicating that POD and PPO enzymes degrade PAHs rapidly under these conditions.

POD and PPO exerted different effects in terms of degradation of PAHs *in vitro*. Rapid decomposition of PHE and ANT by POD occurred. The degradation fraction increased sharply to 17.2% and 26.3% within the first 20 min, and thereafter approached a steady state from 20–60 min. No apparent difference in NAP degradation in the first 30 min was observed for POD *vs*. the POD-free control, but NAP degradation increased slightly to 3.53% from 30–60 min. Clearly, POD degraded PHE and ANT more rapidly than NAP. In contrast, degradation of the three PAHs by PPO exhibited a linear increase; up to ∼18% was degraded after 60 min. No apparent differences in degradation rates or magnitudes were found among the three PAHs, indicating that PPO is not selective in terms of its degradation of the studied PAHs.

### Effects of AA on POD and PPO activities in roots

POD and PPO activities in tall fescue roots were measured in the presence and absence of AA for 16 days (Figure 2). Activities decreased over time irrespective of the presence of AA. For instance, POD and PPO activity in the roots of the control treatments was 1.11 and 0.59 katal/mg root, respectively, at day one, but decreased to 0.58 and 0.47 katal/mg root, respectively, at day 16. Similar decreases in enzyme activity over time also occurred in the presence of AA. However, the addition of AA led to marked reductions in enzyme activities in plant roots. During the study period (16 days), POD and PPO activities in roots in the presence of AA were approximately 26.5–83.2% and 29.1–89.1% lower, respectively, than those of the controls. Furthermore, the presence of AA resulted in rapid decreases in enzyme activity during the first 8 days, after which a relatively steady state was maintained.in 8–16 days. On day 16, the POD and PPO activities in the presence of AA were 0.10 and 0.05 katal/mg root, respectively. Therefore, 2.0 mg/L AA inhibited the POD and PPO activities in plant roots.

The reductions in enzyme activity were estimated by calculation of E_enzyme_ (%):
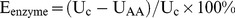
(2)where U_AA_ and U_c_ are the enzyme activities in plant roots grown in the presence and absence, respectively, of AA. A greater E_enzyme_ value indicates greater inhibition of enzyme activity. Figure 3 shows the E_enzyme_ values of the test enzymes in roots as a function of time. The E_enzyme_ values of both POD and PPO, increased dramatically with time, indicating that AA inhibited both enzymes. AA generally inhibited PPO to a greater extent than POD during the study period.

### Effects of AA on the enhanced accumulation of PHE in roots

The impact of AA on root PHE uptake (as a representative PAH) was evaluated using the results of the hydroponic experiments. The PHE concentration decreased over the exposure period (Figure 4a), which may be due to either PHE degradation or metabolism by the plant. Addition of AA enhanced PHE accumulation in the roots of tall fescue. The corresponding PHE concentrations in AA-treated plants were 24.3–44.5 mg/kg, being 27–313% greater than those (5.88–35.0 mg/kg) of the control. In addition, the PHE concentration in AA-treated plant roots decreased rapidly during the first 8 days, and thereafter approached a steady state at day 8–16. This is consistent with the AA-mediated inhibition of enzyme activity over time (Figure 2).

Figure 4b shows the accumulated mass (M_PHE_, μg) of PHE in tall fescue roots as a function of uptake time. M_PHE_ was calculated by multiplication of the concentration of PHE in roots (C_PHE_, μg/g) and plant root mass (M_root_, g).
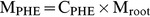
(3)Similar to PHE uptake in roots, PHE accumulation decreased over time. Plant growth results in an increase in biomass that may dilute PHE concentration, particularly during rapid growth. PHE mass is a better descriptor of solute uptake because it excludes the effect of dilution. The presence of AA significantly enhanced PHE accumulation by tall fescue roots (Figure 4b). As evidenced by the reduced PHE uptake in its absence, the presence of AA inhibits plant enzyme activity, and so enhances PHE accumulation in tall fescue roots.

The dissipation of organic compounds in plants is due to the metabolic processes therein [11], [18], [19]. The presence of AA inhibits such metabolic processes, leading to enhanced PAH accumulation. We quantified this effect by means of inhibition efficiency (E_PHE_, %):
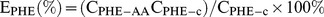
(4)where C_PHE-c_ and C_PHE-AA_ represent the PHE concentrations in plant roots grown without and with AA, respectively. A higher E_PHE_ value indicates greater inhibition of PHE metabolism. The E_PHE_ values increased from 27% to 313% from days 1 to 16 (Figure 5), indicating that the presence of AA persistently inhibited enzyme activities and hence PHE metabolism in plants. Such inhibitory effects became more significant with increasing exposure time.

## Discussion

Our data, together with several recent reports, indicate that PPO and POD facilitate the decomposition of PAHs and other aromatic hydrocarbons [25], [26], [27]. Moen *et* *al*. (1994) first reported metabolism of PHE by manganese peroxidase (MnP) in *Phanerochaete chrysosporium* in a lipid peroxidation-dependent process [37]. MnP from *Nematoloma frowardii* degraded ANT, PHE, pyrene, fluoranthene, and benzo[a]pyrene, leading to the partial mineralization of these persistent compounds [38], [39]. The MnP of *Irpex lacteus* efficiently decomposed 3- and 4-ring PAHs including PHE and fluoranthene [40]. *Irpex lacteus* in whole fungal cultures could transform ANT and open its aromatic rings *in vivo*, for which MnP is the essential enzyme [41].

In our study, POD preferentially decomposed ANT and PHE rather than NAP *in vitro*. This is in contrast to the dogma that larger fused-ring PAHs are more resistant to degradation. PPO decomposed PAHs, but was not specific for a particular compound. POD and PPO are large proteins (33–200 kDa for PPO, and 35–105 kDa for POD) [25], [42]. The catalytic degradation of organic chemicals by enzymes depends on the enzyme structure and complex formation. Henriksen *et* *al*. (1999) reported that horseradish peroxidase forms peroxidase and ferulic acid binary complexes and peroxidase, cyanide and ferulic acid ternary complexes, which contain the flexible aromatic donor binding sites. The complex component ferulic acid is a naturally occurring phenolic compound commonly found in plant cell walls [43]. The flexible binding sites may interact with organic chemicals such as PAHs and initiate decomposition. The more hydrophobic nature of PHE and ANT and their relatively large size might lead to stronger binding sites than that of NAP; this is presumed to be responsible for the preferential decomposition of PHE and ANT. Exotic organic contaminants (*e*.*g*., PAHs) in plants also impact enzyme activity [34]. The relatively high concentration of NAP (10 mg/L) used might have reduced POD activity, leading to negligible NAP degradation by POD at 0–30 min and only slight degradation at 30–60 min. Overall, the mechanism of decomposition, and particularly the selectivity of the POD enzyme, remains unclear, and is worthy of further study.

Organic contaminants in plants may be partially metabolized, leading to reduced concentrations in foods and lowering the risk of their consumption by humans. Enzyme types and activities are believed to control the metabolism and degradation of these contaminants in plants [22], [23]. PAHs in plants could be metabolized to form more polar products. Wild *et* *al*. (2005) reported the degradation of ANT in the cortex cells of maize (*Zea mays*) and wheat (*Triticum aestivum*) using two-photon excitation microscopy [16]. Zhang *et* *al*. (2010) reported that the major metabolites of ANT in ryegrass were anthraqinone and anthrone [44]. The former was further metabolized, while the latter accumulated. However, little information on the role of enzyme activity on PAH decomposition is available. We demonstrate here that POD and PPO degraded PAHs (Figure 1).

Enzymes control the decomposition and metabolism of organic chemicals in plants [22], [23]. AA uptake inhibited enzyme activity and hence diminished PAH metabolism by plants. As a result, enhanced PAH accumulation is expected. Plotting of E_PHE_ values against the E_enzyme_ values of POD and PPO (Figure 6) showed a significant and positive correlation between E_PHE_ and E_enzyme_, which further confirms that AA inhibits POD and PPO activities, reducing PHE degradation. This results in enhanced PHE accumulation in tall fescue roots and higher risk of contamination of the ecosystem. To further confirm this, tall fescue roots were immersed in water at 100°C for 5 min to inhibit enzyme activity. The hot water-treated plants and untreated tall fescue were then exposed to various PHE concentrations. The PHE concentration in roots was measured at 12, 24, and 48 h (Figure 7). The PHE concentration in hot water-treated roots (enzyme activity inhibited) was significantly higher than that in untreated roots, confirming that inhibition of enzyme activity results in enhanced PHE accumulation in roots.

Our data suggest that the use of external chemicals (*i*.*e*., AA) to regulate enzyme activity in plants influences PAH accumulation. This suggests that the application of enzyme inhibitors enhances the PAH-contamination risk. Many commonly utilized inhibitors may influence enzyme activity and hence impact the metabolism of organic chemicals by plants. In this study, AA was shown to inhibit PPO and POD activities, and consequently increase PHE accumulation, in plants. In general, PHE accumulation depends on plant uptake capacity, volatilization, dilution due to growth, and metabolism. PAH uptake by plants is a series of passive partition processes [9], and there was no apparent difference in biomass irrespective of the presence of AA. The volatilization of PHE from plants makes a minor contribution (if any) due to its relatively low KH [45]. Thus, the difference in PHE accumulation in roots in the presence and absence of AA (Figure 4) was likely due to altered enzymatic PHE degradation. The positive relationship between E_PHE_ and E_enzyme_ (Figure 6) indicates that AA inhibits enzymatic PHE degradation, leading to greater residual PHE in plants.
